# Effects of Onion Juice on the Normal Flora of Eyelids and Conjunctiva in an Animal Model

**DOI:** 10.5812/jjm.9678

**Published:** 2014-05-01

**Authors:** Mahmoud Nejabat, Alireza Salehi, Parisa Noorani Azad, Mohammad Javad Ashraf

**Affiliations:** 1 Research Center for Traditional Medicine and History of Medicine, Shiraz University of Medical Sciences, Shiraz, IR Iran; 2 Department of Pathology, Shiraz University of Medical Sciences, Shiraz, IR Iran

**Keywords:** Onions, Conjunctiva, Eyelids, Anti-Infective Agents, *Staphylococcus lugdunensis*, Medicine, Traditional

## Abstract

**Background:**

Traditional medicine/complementary alternative medicine may suggest new ideas to modern medicine in order to face new challenges however these concepts should be acknowledged based on experimental studies.

**Objectives:**

We aimed to study the effects of onion (*Allium cepa*) juice on the normal flora of conjunctiva and eye lids, and to follow the histopathology changes of conjunctiva in an animal study.

**Materials and Methods:**

Twenty-four rabbits were randomly classified into three equal groups. Groups 1, 2 and 3 received fresh red onion juice, as an eye drop, into the right eye twice daily for; one week, one month, and two months, respectively. Microbiological sampling by sterile swabs was performed before and after the intervention. Cultural characteristics, including the growth rate and the kind of organisms, are reported. At the end of the study, pathological samples were collected from the inferior fornix.

**Results:**

After the intervention, the number of positive cultures in the samples, collected from both the conjunctiva and eyelid, had decreased. Group 3 demonstrated the lowest amount of growth after the administration of the onion juice and the bacterial isolation rates from each organism had decreased. All pathological samples revealed some degree of inflammation. There was no evidence of metaplasia or dysplasia. There was no significant difference between the growth rates of organisms in the experimental groups using statistical analysis.

**Conclusions:**

According to our experiment, onion has an inhibitory effect on the growth of normal eye flora; although the duration of onion juice instillation did not show any significant effect on the group results. Hence, this finding is an initiating point for further investigations into the antimicrobial properties of this herb to treat common eye infections, including conjunctivitis and blepharitis.

## 1. Background

With regard to the progress of modern medical approaches, a new logic-based combination of modern medicine, with existing facilities in the fields of traditional medicine, has resulted in complementary medicine. Thus, modern medicine will face new challenges using valuable and harmless alternative methods based on complementary medical findings.

In times past, traditional medicine/complementary alternative medicine (TM/CAM) played a special role in the treatment of eye diseases. Moreover, ophthalmology was paid a great deal of attention in traditional literature, especially in Iranian traditional medicine. Among old civilizations, unproven treatments were used to treat different types of ophthalmological diseases, including; injecting cobra venom for macular degeneration, and biogenic stimulation with tissues such as placenta, injected sub conjunctivally for incurable eye diseases (1). New CAM approaches in ophthalmology employ various methods, including; acupuncture, dietary supplements, herbal remedies and homeopathy.

In Iranian traditional medical literature, the medicinal values of onion (*Allium cepa*) are frequently mentioned. Avicenna (980 – 1037 AD), in the ‘Canon of Medicine,’ brought detailed discussions of ophthalmic disease, such as the diagnosis and treatment of cataracts (2), and noted the beneficial effects of onion on the eye. As described, ‘the extract of edible basl (onion) is useful for cataracts and clears vision, collyrium of powdered seeds with honey is useful in corneal opacity’(3).

About two centuries ago in,‘Makhzan-O-L Advieh,’a famous Iranian traditional pharmacopoeia, written by Mohammad Hossein Aghili Khorasani Shirazi (d. 1772 AD), one of the most significant Persian physicians, onion was prescribed for the prevention and treatment of a number of eye diseases, including; cataract, epiphora and blepharitis (4).

Onion is one of the oldest cultivated plants, and it has been used for many centuries as a natural flavoring, herbal medicine, and among some civilizations it even hadreligious connotations (5). Onions contain; allins, flavonoids, steroid saponins, polysaccahrides, fructosans, saccharose and other sugars (6). These compounds have strong antioxidant, antibacterial and anti-inflammatory properties (5-7). Thiosulfinates are the best studied volatile compounds arising from alliums. In addition to its pungency and flavoring characteristics, it also exhibits antimicrobial effects (6). Flavonoids, are another compound of onions, and their significant antioxidant properties inhibit free-radical mediated events in cells that lead to tissue inflammation (7-9). Steroid saponins are another important component, which have medicinal value and these have been studied in animal models (10).

Unfortunately, antibiotic resistance continues to increase. *Streptococcus pneumonia* and *Staphylococcus aureus*are the most common ocular pathogens that quickly develop resistance to ophthalmic antibiotics (11). Therefore, novel compounds as alternative agents are critically needed. One of the recent approaches is to focus on traditional herbal medicine in order to develop new medications. 

## 2. Objectives

To the best of our knowledge, there have been no previous studies on the antibacterial activity of onion, on ocular conditions. Thus, this study was carried out to explore the antibacterial effects of onion juice against the normal flora that inhabits the conjunctiva and eyelid surfaces, and to assess further histopathologic changes. 

## 3. Materials and Methods

A total of 30 white Dutch rabbits, aged between 6 and 18 months, with a mean weight of 2200 ± 500 g, were selected for the study. In the initial screening, the animals were examined carefully and any that had symptoms of eye problems, such as; redness, inflammation, chemosis, conjunctivitis, mucopurulent discharge, or any other ophthalmic or general clinical disease such as any evidence of upper respiratory tract infection, were excluded from the study.

Experiments were conducted in accordance with the ARVO Statement for the Use of Animals in Ophthalmic and Vision Research and the Guiding Principles in the Care and Use of Animals. The rabbits were divided into three groups. All of the animals were kept in a room containing only rabbits. Two rabbits were each housed in one cage and there was no direct connection between the cages. They were fed daily with commercial pet food, which was placed in front of the cages. The rooms were appropriately ventilated. Prior to any intervention, animals were anesthetized with ketamine 50 mg/kg to provide appropriate conditions.

Conjunctival and eyelid swabs were collected from the right eye of the rabbits at the beginning of the study, before the intervention. Sterile cotton swabs were moistened with sterile normal saline which werethen wiped along the lower fornix of the right eye by everting the eyelid. During sampling, any contact of the swabs with eyelashes, eyelid skin or lacrimal punctum was avoided. A separate sterile swab was used for each eyelid specimen. Then the swabs were immediately streaked onto a blood agar plate. The plates were incubated at 37ºC for 24 hours. After 24 hours, the cultures were examined. If there was not enough growth of the colonies, they were reincubated for a further 24 hours. After 48 hours, the isolated colonies were identified by cultural characteristics and gram staining. By performing this phase, before instillation of the onion juice, we were able to find out more about the most common organisms that frequently inhabit the conjunctiva and eyelid of healthy rabbits. These results were then used as control data to compare with the results obtained after onion juice administration.

A standard fluorescein strip was applied to the eye for cornea and conjunctival staining. The areas of positive staining showed epithelial changes. Any positive finding was recorded on a data sheet. According to previous reports, red onion has the highest content of flavonoids among other kinds of onions (12). Therefore, fresh red onion was purchased from local markets. The onion juice was prepared fresh each time before instillation, by peeling and grinding the onion for about 10–15 minutes using a blender. Then the ground material was filtered by Nalgene sterile bottle filters, and the crude extract centrifuged (3000 rpm, at 4°C) until a relatively clear supernatant liquid was obtained. The onion juice was then poured into a sterile eye dropper.

Twice daily, the juice was instilled into the right eye of the animals. Group 1 received onion juice eye drops for one week, Group 2 for one month, and Group 3 were exposed to the juice for two months. At the end of the study, a microbiological assessment of each eye’s normal flora was conducted using the same method described in the preliminary phase and a pathological specimen was obtained from the lower conjunctival fornix of the right eye in the form of a tissue strip (about 5-6 mm^2^). They were kept in formalin as a transport medium. 

After sample processing, very fine sectional specimens embedded in paraffin were cut and then stained with hematoxylin and eosin (H&E) to determine the presence or absence of; inflammation, metaplasia, dysplasia and fibrosis. The pathologist, who performed all of the histological examinations, was blinded to the groups. Chi-squared tests were used to analyze the data and compare the results between the three studied groups. A P value less than 0.05 was considered as significant.

## 4. Results

Of the 30 rabbits initially examined, six were excluded because they had evidence of ophthalmic or respiratory disease. A total of 24 rabbits were divided into three groups consisting of eight animals. Group 2 had a lower median age. Bacteria were isolated from 66% (16 out of 24) of conjunctival swabs. *Staphylococcus*, *Bacillus* and *yeast species *were the most commonly isolated organisms. *Staphylococcus*, *Bacillus*, *Proteus*, *Pseudomonas*, *Escherichia coli*, *yeast *and fungal species were found in the conjunctiva samples.

Among the 24 eyelid samples, 22 swabs (91.6%) had positive cultures. Cultivated isolates included; *Staphylococcus*, *Bacillus*, *Proteus*,* Pseudomonas, Enterobacter *and *fungal species*. *Staphylococcus*, *Proteus *and *Bacillus* were the most frequent colonies found. Table 1 shows the frequencies of organisms from positive cultures of conjunctiva and eyelid swabs before the intervention.

**Table 1.** Frequencies of Organisms With Positive Cultures Collected From Conjunctiva and Eyelid Surface Before the Intervention

Organism	Frequency of Conjunctival Isolation ^a^, No. (%)	Frequency of Eyelid Isolation ^a^, No. (%)
***Staphylococcus*** **spp.**	7 (29)	19 (79)
***Bacillus*** **spp.**	4 (16)	4 (16)
***Pseudomonas *** **spp.**	2 (8)	1 (4)
**Proteus**	2 (8)	6 (25)
***Escherichia coli***	1 (4)	0 (0)
***Enterobacter*** **spp.**	0 (0)	1 (4)
**Yeast ** ***spp.***	5 (20)	0 (0)
**Fungal *spp.***	4 (16)	3 (12.5)

^a^From 24 swabs

Rates of colony growth were reported in three ranges; mild growth (1-10 colonies), moderate growth (11-50 colonies), and heavy growth (> 50 colonies). After the intervention, the frequencies of positive cultures decreased to 33.3% in the conjunctival swabs and to 79% in the eyelid samples. The frequency of microorganisms found in the conjunctival swabs was reduced in most of the cases. Positive cultures of *Staphylococcus* and *Bacillus *diminished from 29% to 8% and from 16% to 4% (out of the total 24 conjunctiva samples), respectively. Of the four cases of fungal isolates in the conjunctival swab samples, all were eliminated after the intervention. In addition, no isolates of *Pseudomonas*, *E. coli*, *Proteus*, or yeast were observed after the intervention in the positive conjunctival cultures.

**Figure 1.**
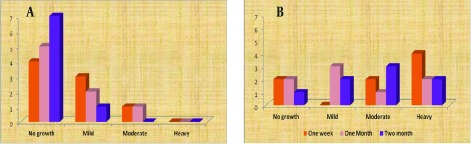
Growth Rates of Eyelid and Conjunctival Cultures Afterthe Intervention in Different Groups (a) Conjunctiva, (b) Eyelid

**Figure 2.**
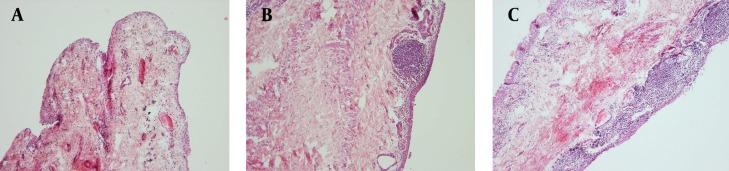
Photographs of Pathological Specimens Showing conjunctive inflammation, (H&E) staining ×100: A) mild inflammation; B) moderate inflammation with fibrosis; C) severe inflammation.

Among cultivated isolates after onion juice instillation, only two new isolates were found. Table 2 shows the frequencies of organisms from positive cultures of conjunctiva and eyelid swabs after the intervention.Most of the negative conjunctival cultures after onion juice administration belonged to Group 3; although no statistical significance was detected between the groups’ results (P = 0.25). The rate of growth of the eyelid and conjunctival cultures after the intervention in the different groups are shown in Figure 1. Histological examinations of all conjunctiva slides revealed some degree of inflammation (Figure 2). Group 3 had the highest level of inflammation compared to the other two groups (P = 0.045). No metaplasia or dysplasia was detected among the tissue samples. Scar formation and the ratio of goblet cells to epithelial cells showed no significant differences in any of the groups, and no clear relationship was found between them and the severity of tissue inflammation.

**Table 2.** Frequencies of Organisms with Positive Cultures of Conjunctiva and Eyelid Swabs After the Intervention

Organism	Frequency of Conjunctival Isolation ^a^, No. (%)	Frequency of Eyelid Isolation ^a^, No. (%)
***Staphylococcus*** ** spp.**	2 (8)	13 (54)
***Bacillus*** **spp.**	1 (4)	3 (12.5)
***Pseudomonas*** **spp.**	-	-
***Proteus***	-	3 (12.5)
***Escherichia coli***	-	1 (4)
***Enterobacter*** ** spp.**	2 (8)	1 (4)
***Yeast* spp.**	-	-
*** Fungal* spp.**	-	-
	-	5 (20)

^a^ From 24 swabs

## 5. Discussion

Nowadays, with regard to the increasing prevalence of antibiotic-resistant bacterial infections, there is a greater need for alternative treatments. Thus, new approaches for finding novel compounds are needed. One of the recent approaches focuses on traditional herbal medicine in order to develop new medications. Onion (*Alium cepa*) is one of the herbs that have medicinal value, which has often been discussed in traditional literature, and it has also been scientifically studied in recent decades. Studies in this area can be roughly divided into two categories; those which are done *in vitro* and investigate the antimicrobial properties of onion, and those that evaluate the effects of onion juice on ophthalmic conditions. This study was the first project conducted to investigate the antimicrobial effect of onion juice on the eyes of live models.

In comparison to previous studies, our study was conducted to identify the normal conjunctive flora of healthy rabbits, which was found to be similar in most cases. More specifically, our findings are closer to the study carried out by Okuda and Campbell (1974) on 54 New Zealand white rabbits (13). They have realized Bacillus species as the most common isolated organism whereas in the present study the most common isolated organism was *Staphylococcus*. It should be mentioned that our findings were compatible to a study by Cooper et al. in terms of type of microorganism (14). The sampling and isolation methods used were similar to the Cooper study, except that distilled water was used in their study. According to the results, the number of positive cultures declined in both the conjunctival and eyelid samples after the instillation of onion juice. *Pseudomonas*, *E. coli*, *Proteus*, yeast, and other fungal species, were not detected in conjunctival swabs after the intervention. The highest inhibited growth was observed in *Staphylococcus* colonies.

The above results are consistent with previous studies carried out to investigate the antimicrobial effects of onion. For example, Chen et al. suggested that onion could inhibit the growth of *Pseudomonas aeruginosa*, *E. coli*, *Bacillus cereus*, *S. faecalis* and *P. vulgaris *(15). Furthermore, Kim et al. in 2004 found onion oil exhibited high anti-yeast activity showing a minimal inhibitory concentration (MIC) of 40 ppm in soy sauce (16). Despite the differences between the descriptive statistics of the studied groups, chi-squared analysis demonstrated that no significant difference existed in the growth of organisms (P = 0.25). This fact may have resulted from limitations in the number of animal models. Yet the duration of onion juice administration could not be concluded to be a decisive factor on the group results.

The inhibitory effect of onion juice on the growth of conjunctiva normal flora was higher than the eyelids isolates. This may have resulted from the route of administration of the onion extract which was in the form of eye drops. Applying the active onion contents in the form of ointment may cause greater retardation in the growth of eyelid colonies. Pathological samples revealed some degree of inflammation. This finding was in contrast to previous studies which reported on the anti-inflammatory features of onions. Hence, further investigations may be required to clarify the histological changes induced by onion juice on the conjunctiva. There was no evidence of metaplasia or dysplasia. Corneal and conjunctival staining showed no epithelial damage after the intervention. Thus we can tentatively conclude that onion is a safe compound.

Overall the results indicated that onion has an inhibitory effect on the growth of normal flora on conjunctiva and eyelid surfaces. So, it appears that onion juice in the form of eye drops can serve as a new antimicrobial therapy for ophthalmic diseases in future studies and our study may be a starting point for further investigations on this herb.
